# Protective Factors Against e‐Cigarette Use Among First Nations People Aged 16–24 in the Next Generation Youth Wellbeing Study

**DOI:** 10.1002/hpja.951

**Published:** 2025-02-06

**Authors:** Christina L. Heris, Simon Graham, Robyn Williams, Emily Banks, Aryati Yashadhana, Katiska Davis, Justine Whitby, Ted Fields, Michelle O'Leary, Rebecca Ivers, Bette Liu, Christopher D. McKay, Francine Eades, Lina Gubhaju, Tabassum Rahman, Grace Joshy, Sandra Eades

**Affiliations:** ^1^ Yardhura Walani National Centre for Aboriginal and Torres Strait Islander Wellbeing Research, National Centre for Epidemiology and Population Health Australian National University Canberra Australia; ^2^ Poche Centre for Indigenous Health, Faculty of Medicine and Health University of Sydney NSW Sydney Australia; ^3^ Health Sciences, Medical School Curtin University Western Australia; ^4^ Centre of Epidemiology for Policy and Practice, National Centre of Epidemiology and Population Health Australian National University Canberra Australia; ^5^ School of Population Health University of New South Wales Sydney Australia; ^6^ Area Director Aboriginal Health East Metropolitan Health Service Perth Western Australia; ^7^ Centre for Epidemiology and Biostatistics, School of Population and Global Health University of Melbourne Victoria Australia

**Keywords:** Aboriginal and Torres Strait Islander people, e‐cigarettes, First Nations, Indigenous, tobacco, vaping, young people, youth

## Abstract

**Issue Addressed:**

Adolescent e‐cigarette use is increasing and is associated with subsequent smoking. This study examines potential protective factors associated with not vaping among First Nations adolescents in Australia to inform community programs.

**Methods:**

The ‘*Next Generation: Youth Wellbeing Study’* is a cohort study of First Nations adolescents aged 10–24 years from urban, rural and remote communities in Central Australia, Western Australia and New South Wales. Analysis of self‐reported vaping from 16 to 24‐year‐olds, collected 2018–2020, using multi‐level mixed‐effects Poisson regression to estimate age‐site‐adjusted prevalence ratios (PRs) for never‐vaping in relation to various factors.

**Results:**

Among 419 participants, 65% were female, 75% had never vaped, 49% had never smoked and 82% lived in smoke‐free homes. Never vaping was more common among those who had: never‐smoked (PR = 1.78, 95%CI: 1.56–2.04); never used cannabis (1.89, 1.60–2.24); non‐smoking friends (1.38, 1.26–1.51); good mental health (1.15, 1.01–1.30), never diagnosed with depression (1.21, 1.01–1.46) or anxiety (1.31, 1.08–1.57); and no experiences of racism (1.21, 1.08–1.36), no negative criminal justice system experiences (1.25, 1.11–1.41), or vicarious racism through negative media (1.24, 1.10–1.39).

**Conclusions:**

Most First Nations adolescents have never vaped, with potential protective factors being better mental health, no other substance use and fewer experiences of racism and justice system interactions. Comprehensive community adolescent prevention programs are needed to prevent vaping and protect future health, including preventing nicotine addiction and future smoking.

**So What?:**

Policies and programs must address e‐cigarettes directly as well as structural factors, promoting broader adolescent wellbeing, centring culture and family in a strengths‐based approach.

**Abbreviations**
CA: Central Australia
CI: Confidence Interval
First Nations: Aboriginal and Torres Strait Islander Peoples
NHMRC: National Health and Medical Research Council of Australia
NSW: New South Wales
PR: Prevalence Ratio
WA: Western Australia

## 1 Introduction

Electronic cigarettes (e‐cigarettes or vapes) are battery operated devices used to heat ‘e‐liquids’ to produce an aerosol that is inhaled or ‘vaped’ [1, 2, 3]. These liquids commonly contain nicotine. Since 1 October 2021, Australians required a prescription to legally access nicotine e‐cigarettes; [3] however, e‐cigarettes remain accessible (illegally), including to adolescents [4, 5]. From 1 July 2024 e‐cigarettes could only be sold by pharmacies (with a prescription required for those under 18 years) [3], https://www.health.gov.au/topics/smoking‐vaping‐and‐tobacco/about‐vaping#:~:text=smoking%20and%20vaping.‐,Vaping%20and%20e%2Dcigarette%20laws%20and%20reforms,smoking%20or%20manage%20nicotine%20dependence.

E‐cigarettes are often promoted for smoking cessation, including by industry, despite the lack of medically registered e‐cigarette products and issues with establishing the balance of harms and benefits for this purpose. The Royal Australian College of General Practitioners recommends that e‐cigarettes can be used as late‐ or last‐line treatments for smoking, including for First Nations people [6, 7]. However, the tobacco and e‐cigarette industry has targeted First Nations communities and leaders with ‘harm reduction’ claims in an attempt to influence more favourable policy decisions [8].

Evidence is building that e‐cigarettes carry significant harms, particularly for non‐smokers and youth [1, 9, 10]. Nicotine addiction, burns, poisoning, inhalation toxicity and lung injury are some of the established risks of vaping, while for important major conditions such as cancer, cardiovascular disease and respiratory health (apart from lung injury) their effects are not known—meaning that their safety for these outcomes is not established [9, 10, 11]. Newer generation disposable devices, heavily marketed and targeted towards adolescents, expose the user to very high doses of nicotine and increase susceptibility to nicotine addiction, which is a significant risk to adolescent brain development [9, 10]. Especially concerningly for adolescents, non‐smokers/never smokers who used e‐cigarettes are three times as likely to become regular smokers than non‐users [9, 10]. In Australia, smoking remains the largest contributing risk factor to the gap in disease burden between Aboriginal and Torres Strait Islander people and non‐Indigenous Australians [12], and accounts for half of all deaths in adults aged 45 and older [13]. As well as the risks associated with vaping itself, widespread vaping uptake is likely to undermine efforts to reduce tobacco use and smoking harms.

Youth e‐cigarette use is rapidly increasing and is a recognised public health issue. In 2022/23, 48.8% of 18–24 year olds had ever used e‐cigarettes and 20.6% currently used [14]. There is a lack of recent First Nations data, but prevalence also appears to be increasing and more common than in the total population [15, 16, 17].

Identifying factors that help prevent e‐cigarette use (and subsequent tobacco smoking) is important for designing appropriate prevention programs. While some recent studies have identified protective factors against tobacco [18] and cannabis use among First Nations students [19], to date there are no studies exploring the protective factors against e‐cigarette use among older First Nations adolescents and young adults in Australia.

Evidence on tobacco and cannabis indicates likely shared risk or protective factors relevant for e‐cigarettes such as: the influence of family and friends in normalising behaviours; positive family relationships; life stressors (including stable housing, justice interactions), mental health issues and other substance use [18, 19, 20, 21]. First Nations adolescents are more likely to experience these and additional risk factors, due to the ongoing legacy of colonisation, including intergenerational trauma, and contemporary colonisation processes. This includes experiences of racism, discrimination and marginalisation, and systemic barriers to employment and study opportunities [22]. Some general population studies have identified being exposed to e‐cigarette promotion [23], poorer mental health (as both a risk factor for use and an outcome) [24], peer behaviours and attitudes [5, 24], tobacco use [5] and parental smoking [24] or vaping [5], as key factors related to vaping along with curiosity, positive perceptions and interest in flavours [5].

This study aims to identify the protective factors against e‐cigarette use among First Nations adolescents to inform the design of prevention programs for both e‐cigarette use and smoking.

## 2 Methods

### 2.1 Study Design—Next Gen Study

The ‘Next Generation: Youth Wellbeing Study’ is a mixed‐methods cohort study of First Nations adolescents aged 10–24 years from urban, regional and remote communities in Central Australia (CA), Western Australia (WA) and New South Wales (NSW) [25]. This study uses the term ‘adolescents’ to mean 10–24 year olds [26].

### 2.2 Recruitment

Recruitment has been described elsewhere [25, 27]. Briefly, 1244 First Nations participants aged 10–24 years were successfully recruited in family groups by the Aboriginal research team through trusted community networks (personal contacts, through Aboriginal community organisations, sports clubs and youth centres) and peer recruitment [27]. This facilitated culturally safe engagement with adolescents and community. Each of the three sites employed dedicated community‐based researchers (including RW, TF, MO) and young Aboriginal peer researchers (KD, JW) who worked to identify and recruit eligible young people and their carers, conducted data collection and chose the incentive in consultation with local partners (e.g., retail vouchers). Once trust had been established large numbers of young people were recruited over short periods (e.g., 200 participants in 3 days) [27].

### 2.3 Data Collection/Administration

Participants independently completed a questionnaire (REDCap survey on tablets) or with some assistance from the research team if requested. Data were collected between 1 March 2018 and 30 March 2020. There were two different age‐appropriate consent processes and questionnaires for participants aged 10–15 years and 16–24 years. This study only uses the 16–24 years data as the 10–15 years questionnaire did not ask about vaping due to ethical and community approvals.

### 2.4 Measures

The main outcome of interest was ‘never used e‐cigarettes’ (vs ever) from the question “*Have you ever tried any of the following: e‐cigarettes (vaping)?*”

### 2.5 Exposures

Exposures were selected based on published literature and community consultations. Demographic factors included location, age, gender and smokefree exposures. Sociodemographic factors included education and schooling, socioeconomic factors, housing, future expectations and boredom. Physical health included tobacco and other substance use, relationships, self‐rated health, physical activity and screentime. Mental health measures included depression, anxiety, the Kessler 5 Distress Score (K5) [28], and the Child and Youth Resilience Measure (CYRM‐12—12‐item three‐point‐scale score) [29]. Participants were asked about Culture, connectedness, identity and Indigenous languages. Finally, several measures of ‘systems of exclusion’ of bullying, personal and vicarious experiences of racism and interactions with the justice system.

### 2.6 Statistical Analysis

We present a cross‐sectional analysis of 16‐24‐year‐olds. Each model has been restricted to those with outcome data (never/ever used e‐cigarettes). Missing data for covariate and exposure variables were retained in the analyses as a separate category. Descriptive statistics are provided for the overall sample (number (*n*) and proportion (%)) and for each exposure.

Multi‐level mixed‐effects Poisson regression models estimated prevalence Ratios (PRs) with 95% confidence Intervals (95%CI) and robust standard errors to quantify the association between never vaping and each of the exposures, taking a strengths‐based approach [30, 31]. PRs are adjusted for age (continuous) and state/site (to account for clustering by family in the sample). All analyses were conducted in Stata/SE v16.1.

### 2.7 Ethics

The Next Generation Youth Wellbeing Study is a First Nations‐led study (SE), grounded in, abiding by and respecting local cultural protocols. It has oversight from a governance committee of both First Nations and non‐Indigenous people and has been conducted with Aboriginal Community Controlled Health Services and other community partners.

The authorship group includes those with First Nations lived experience (SE, SG, RW, FE, KD, JW, CM, TF, MO) and with research expertise in First Nations health (all authors), adolescent health (all authors) and tobacco control and e‐cigarettes (SE, EB, CH, TR, GJ). First Nations community members were involved throughout all stages of this study. Priority research questions and selected exposure variables were identified through consultation. Feedback on early descriptive results was adopted, with further feedback on the interpretation of the findings incorporated.

Ethics approvals were received from the Central Australian Aboriginal Human Research Ethics Committee (Ref #16–398), Western Australian Aboriginal Health Ethics Committee (Ref #719), Aboriginal Health and Medical Research Council of NSW (AH&MRC) Ethics Committee (Ref #1255–17), Alfred Health Ethics Committee (Ref #255–16) and the University of Melbourne Medicine and Dentistry Human Ethics Sub‐Committee (ID# 1851155). This paper was approved for submission for publication by AH&MRC.

## 3 Results

### 3.1 Sample

Of the 518 participants aged 16–24 years, 99 (19%) were excluded as their vaping status was ‘missing’ (not asked (*n* = 12), skipped the question (*n* = 76), or responded ‘prefer not to answer’ (*n* = 11)), leaving a final sample of 419 participants. The characteristics of those who were and were not included in the study due to missing outcome data did not differ significantly. Half (56%) of participants were from WA, a third from NSW (34%) and 10% from CA. There were substantially more female participants than males (65% vs. 35%) and 65% were aged 18 years or older (Table 1).

**TABLE 1:** Sample characteristics, e‐cigarette and tobacco use and exposure.

	16–24 years (*N* = 419) %(*n*)
Site	
Central Australia	10.0% (42)
Western Australia	56.1% (235)
New South Wales	33.9% (142)
Age	
16–17	35.3% (148)
18–24	64.7% (271)
Sex	
Female	64.7% (266)
Male	35.3% (145)
Use of E‐Cigarettes	
Ever vaped	25.5% (107)
Never vaped	74.5% (312)
Cigarette Smoking	
Ever smoked	51.1% (211)
Never smoked	48.9% (202)
Current Smoking Status	
Current smoker	29.3% (120)
Never/non/ex‐smoker	70.7% (289)
Smoke‐Free Home	
Non‐smokefree home	17.4% (68)
Smoke‐free home	82.2% (322)

### 3.2 E‐Cigarette Use, Tobacco Use and Exposure

Three‐quarters (74%) of participants had never used e‐cigarettes, half (49%) had never smoked, most were currently non‐smokers (71%, includes ex and never smokers) and lived in smokefree homes (82%) (Table 1).

### 3.3 Regression Analysis

#### 3.3.1 Demographic and Sociodemographic Factors

**FIGURE 1:**
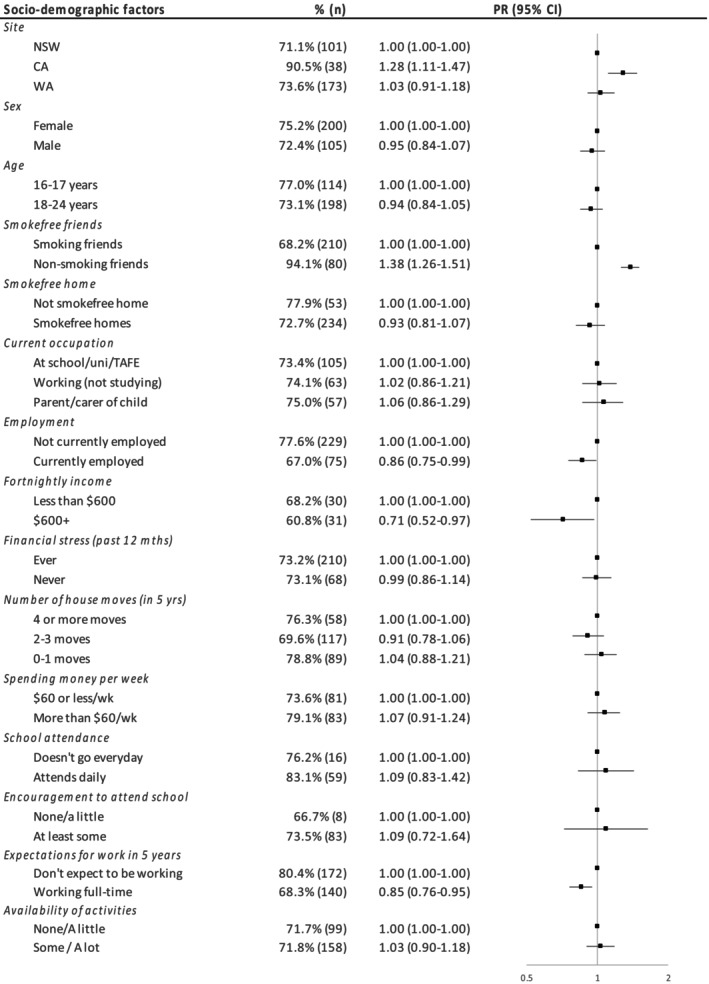
Relationship between demographic/sociodemographic factors and never vaping.

Compared to New South Wales, never vaping was significantly higher in Central Australia (PR = 1.28, 95%CI: 1.11–1.47) and did not differ significantly in Western Australia (PR = 1.03, 0.91–1.18, Figure 1). Having non‐smoking friends was associated with a higher prevalence of never vaping (PR = 1.38, 1.26–1.51). Being currently employed (PR = 0.86, 0.75–0.99), expecting to be working full‐time in the next 5 years (PR = 0.85, 0.76–0.95), and higher income among those currently working ($600+ PR = 0.71, 0.52–0.97) were all associated with lower levels of never vaping.

#### 3.3.2 Physical Health, Mental Health and Cultural Factors

**FIGURE 2:**
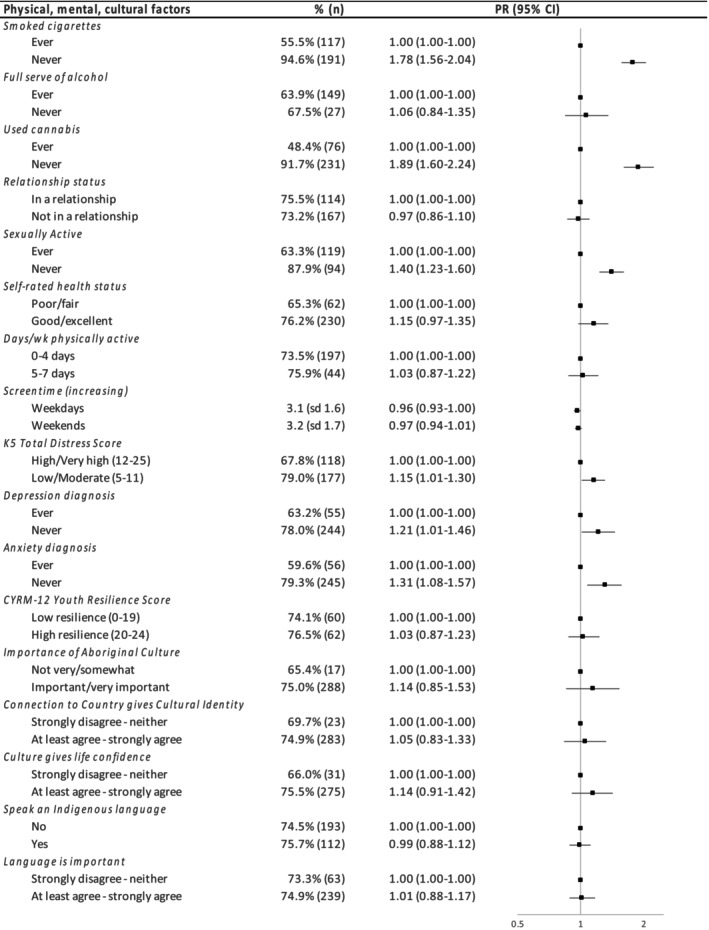
Relationship between physical, mental health and cultural factors and never vaping.

Never vaping was more common among those who had never smoked (PR = 1.78, 1.56–2.04), never used cannabis (PR = 1.89, 1.60–2.24) and not being sexually active (PR = 1.40, 1.23–1.60) (see Figure 2, Table S2), as well as having good mental health (lower psychological distress (PR = 1.15, 1.01–1.30)), never been diagnosed with depression (PR = 1.21, 1.01–1.46) or anxiety (PR = 1.31, 1.08–1.57), compared to not experiencing these exposures. The prevalence of never vaping among those who had never drunk alcohol, with higher self‐rated health, more physically active, with lower levels of screentime and had greater agreement with the cultural factors did not differ significantly from that in other cohort members, noting the relatively wide confidence intervals.

#### 3.3.3 Systems of Exclusion

**FIGURE 3:**
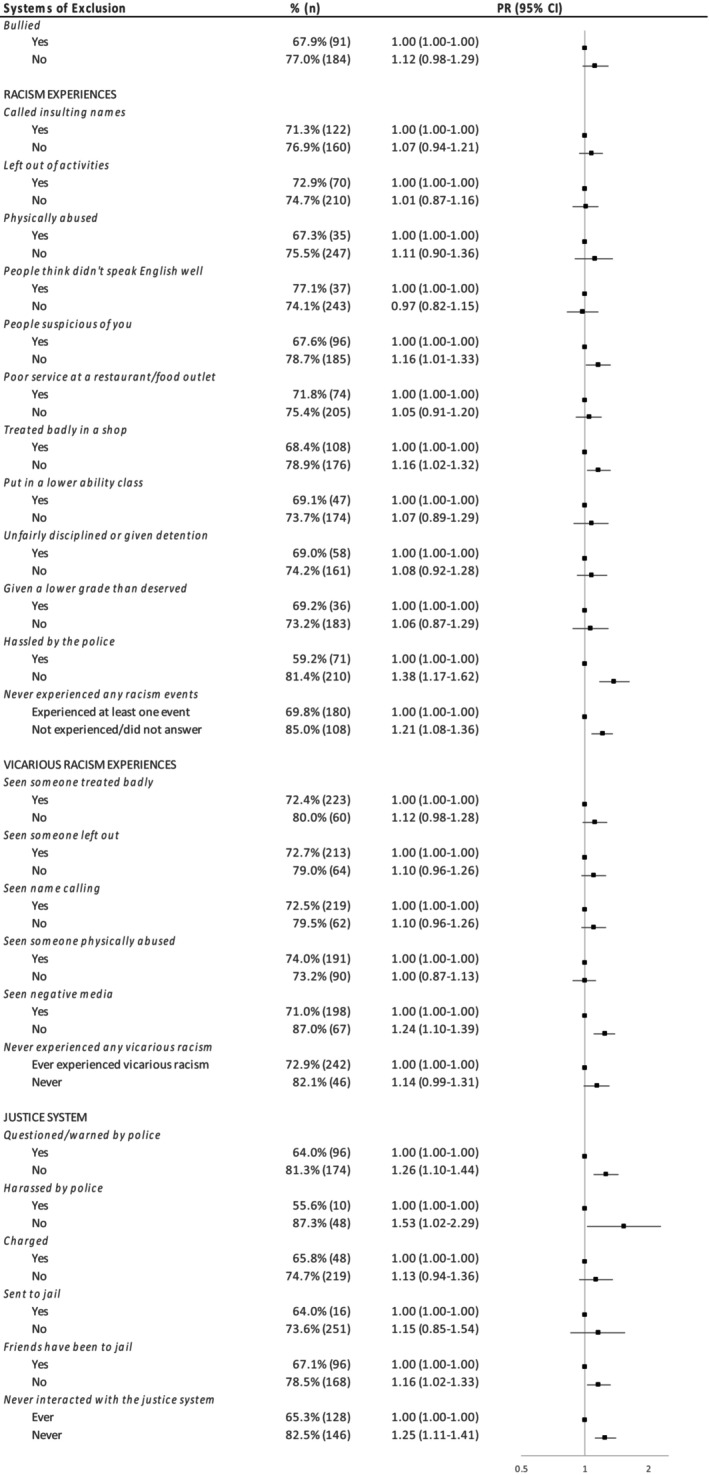
Relationship between systems of exclusion and never vaping.

Never vaping was significantly associated with never personally experiencing any type of racism (PR = 1.21, 1.08–1.36) (Figure 3) and the individual measures: not being hassled by the police (PR = 1.38, 1.17–1.62), treated with suspicion (PR = 1.16, 1.01–1.33), treated badly in a shop (PR = 1.16, 1.02–1.32); and not experiencing vicarious racism in the form of negative media (PR = 1.24, 1.10–1.39). Never vaping was consistently higher for those who had not had interactions with the justice system overall (PR = 1.25, 1.11–1.41), and never questioned (PR = 1.26, 1.10–1.44) or harassed by police (PR: 1.53, 1.02–2.29) or had friends who had been to jail (PR = 1.16, 1.02–1.33), compared to cohort members experiencing these interactions.

## 4 Discussion

This study finds that the majority of First Nations adolescents had never vaped or smoked tobacco and most lived in smoke‐free homes. E‐cigarette use varied markedly by social, behavioural and cultural factors, with lower levels observed in those with better mental health, smokefree influences, no other substance use, experiences of racism or justice system interactions. Our study is the first to identify positive health and wellbeing exposures associated with never vaping among First Nations adolescents in Australia.

In our study, participants living in CA were more likely to have never vaped compared to participants living in urban and regional areas of WA and NSW, with very few (< 5) having ever tried e‐cigarettes. This is in line with national data that most First Nations adults who vaped were in major cities or regional centres, with use rare in remote locations [15]. This is likely due to the wider availability of vaping products in non‐remote areas at the time of data collection, however with the increased availability via online sales and more accessible disposable devices this may have changed. Government reforms are intended to curb this access.

Working in paid employment was associated with a lower prevalence of never vaping, unlike patterns seen with tobacco use [22]. This may relate to the ways in which vaping devices and e‐liquids were predominantly available at the time of survey through vape shops and online stores and the earlier generation of e‐cigarettes (e.g., ‘tanks’) which required some financial investment, compared to the way a packet of cigarettes is shared [22]. It is likely this will also have changed with the widespread proliferation of cheap, disposable vapes [32].

Our study found a strong association between vaping and smoking tobacco, consistent with what has been demonstrated previously with First Nations secondary students in Australia [16], Australian youth generally [16, 17] and other populations [9, 11, 33, 34]. These studies have also found a relationship between vaping and high rates of heavy drinking [34] and other substance use [11]. While we did not find an association with alcohol, there was with cannabis. This clustering of harmful behaviours mirrors that previously shown with tobacco and cannabis with First Nations young people in Australia [19, 21] and highlights the need to address risk behaviours in a comprehensive and holistic way. Within a comprehensive approach there is a need to also increase awareness of the harms of e‐cigarette use among young people, and counter a perception vaping is less harmful than tobacco smoking [35]. There is also an urgent need to combat the direct efforts of the e‐cigarette industry to appeal to young people and generate curiosity, including regulating online advertising and banning flavours [36]. Government reforms, if implemented successfully, will address these issues [37].

Our study found that never vaping was associated with having better mental health. Although some studies have previously explored a relationship between depressive symptoms and e‐cigarette use (and the bi‐directional nature of this relationship) [24, 38] and other mental health conditions [39], a large international review found there was insufficient evidence of a causal relationship between e‐cigarette use and clinical or sub‐clinical mental health conditions [9]. Nicotine is highly addictive and addiction is itself an adverse health condition. Nicotine exposure in any form is detrimental to an adolescent's developing brain and can lead to long term addiction and other mental health conditions [11]. Nicotine cessation may improve mental health symptoms [40]. We found a cross‐sectional association between mental health factors and e‐cigarettes. Again consistent with previous studies with other First Nations young people and tobacco [18] and cannabis use [19], further highlighting the need to address the broader determinants of substance use and the circumstances that create stressful life conditions, and opportunities to support social/emotional resilience.

We found an association between never vaping and not having interacted with police or the justice system. A similar relationship has been shown among Canadian adolescents and young adults [41]. Although it has been illegal to import and purchase nicotine e‐liquids and e‐cigarettes in Australia without a doctor's prescription [3], illicit use is unlikely to be the primary contributor to this relationship as it matches what has been previously demonstrated around tobacco use in First Nations young people and adults [18, 42]. It is likely that this again reflects the broader social determinants of smoking and e‐cigarettes, including systemic racism within the justice system. Not having experienced a range of racism events, personally or vicariously, all trended towards a greater prevalence of never vaping, in some cases significantly, a relationship that has also been found with tobacco use in First Nations adults [43]. The pattern of higher levels of never vaping in the absence of racism, discrimination and negative experiences with the criminal justice system, is consistent with upstream determinants of e‐cigarette use having an impact on health behaviours.

### 4.1 Strengths and Limitations

A key strength of Next Generation is the First Nations leadership, governance and involvement throughout. Trusted relationships were critical for recruitment and will be important in follow‐up. Further, we took a strengths‐based approach [31] to reporting key protective factors against vaping, including a range of positive exposures that are not frequently included in studies of ‘risk behaviours’.

Adolescents were recruited from specific locations, and as a result our sample may not be generalisable to all young First Nations people living in other areas of Australia. This study had a limited sample size which likely impacted the potential to detect statistically significant differences. Those who did not consent to participate in the study may have different risk behaviours or views. Participants self‐reported their vaping status, tobacco use, cannabis use, alcohol consumption, anxiety and depression status. There is potential for under‐reporting of questions associated with negative stereotypes or shame.

The cross‐sectional nature of this study limits assumptions about drivers of e‐cigarette use as we are only able to describe factors associated at the time of survey. Longitudinal studies would provide an opportunity to explore the relationship between the proposed protective factors in preventing uptake over time.

Finally, this is not a prevalence study, and so the levels of never vaping reported in this sample are not necessarily indicative of population vaping prevalence in First Nations adolescents. While our absolute estimates of e‐cigarette use may not be directly representative of First Nations adolescent population in Australia, PRs, which are based on internal comparisons, are still likely to be generalisable [44]. Further, e‐cigarettes were a relatively new product at the time of survey and their availability has increased significantly in recent years [4, 14, 45, 46]. Our data collection (2018–2020) mostly occurred prior to widespread use in Australia and so the lower prevalence in this study does not reflect current use patterns and may have limited our power to detect true relationships. The upcoming Next Generation study follow‐up wave provides an opportunity to explore e‐cigarette use in a contemporary context and understand changes over time.

### 4.2 Implications and Recommendations

Federal and state/territory governments have tightened access and product regulations including banning disposable products from 1 January 2024, with new measures such as banning most flavours, ending retail sales and requiring pharmaceutical‐style packaging for products to be sold through chemists only [37, 47].

There is significant community concern about the rapid proliferation of e‐cigarettes, their use among non‐smokers and the mounting evidence of a longitudinal relationship between vaping and later smoking among young people. Community‐controlled and mainstream smoking cessation programs have supported many First Nations people to successfully quit tobacco, and with evidence‐based resources can similarly support vaping cessation. There is an urgent need to reduce the supply of e‐cigarettes and introduce measures that deter use, including addressing industry tactics such as flavourings and online advertising as well as targeted communication about the risks and harms of e‐cigarette use.

There is also a need for comprehensive holistic programs that support health and wellbeing more generally, including in promoting good mental health, as it is likely this will have an impact on a range of behaviours including smoking, cannabis use and alcohol. While there is a need to address e‐cigarette use specifically, programs should centre family and culture in a strengths‐based, culture‐as‐health approach [48]. While our study did not find an explicit relationship between never vaping and cultural factors, engaging in cultural practices and connection to Country is protective for mental health [49, 50], which is associated with being vape‐free. Further research on culture and language programs as prevention strategies is warranted. However, these programs cannot be expected to be successful without adequate action on the upstream determinants from ongoing colonisation leading to systemic racism, discrimination and the overrepresentation of First Nations people, including children, in the Australian criminal justice system.

## 5 Conclusion

The rapid rise in adolescent vaping is a significant concern. There is a need for specific e‐cigarette information in prevention and cessation programs, reinforced by strong regulation and enforcement banning advertising and flavours that target adolescents. The findings from this study show there are shared risk and protective factors relating to mental health and other substance use and therefore addressing the broader social determinants of health is important. This includes promoting social and emotional wellbeing and addressing structural/systemic issues (such as racism and discrimination, particularly in the justice system) that impact a range of health behaviours including vaping. Programs promoting broader youth wellbeing must centre culture in a strengths‐based approach.

## Conflicts of Interest

The authors declare no conflicts of interest.

## Supporting information


Data S1.

**Table S1.**Relationship between demographic/sociodemographic factors and never vaping.
**Table S2**. Relationship between physical, mental health and cultural factors and never vaping.
**Table S3**. Relationship between systems of exclusion and never vaping.

## Data Availability

The data that supports the findings of this study are available in the supplementary material of this article.
