# Supplementary material for: Protective Factors Against e‐Cigarette Use Among First Nations People Aged 16–24 in the Next Generation Youth Wellbeing Study
Source: Health Promot J Austr. 2025 Feb 6;36(2):e951. doi: 10.1002/hpja.951 (PMC11926581; doi:10.1002/hpja.951)
Supplement: Supplementary file 1 — Data S1. [file HPJA-36-0-s001.pdf]

Supplementary Table 1: Relationship between demographic/sociodemographic factors and never vaping

	Ever Used E-cigarettes % (n)	Never Used E-cigarettes % (n)	Never Used E-cigarettes PR Adj^ (95%CI)
**DEMOGRAPHIC**			
**Site**			
New South Wales	28.9% (41)	71.1% (101)	1
Central Australia	9.5% (4)	90.5% (38)	1.28 (1.11,1.47)*
Western Australia	26.4% (62)	73.6% (173)	1.03 (0.91,1.18)
Missing	0% (0)	0% (0)	
**Sex**			
Female	24.8% (66)	75.2% (200)	1
Male	27.6% (40)	72.4% (105)	0.95 (0.84,1.07)
Missing	12.5% (1)	87.5% (7)	
**Age group**			
16-17	23.0% (34)	77.0% (114)	1
18-24	26.9% (73)	73.1% (198)	0.94 (0.84,1.05)
Missing	0%	0%	
**Friends who smoke**			
Have smoking friends	31.8% (98)	68.2% (210)	1
Non-smoking friends	5.9% (5)	94.1% (80)	1.38 (1.26,1.51)*
Missing	15.4% (4)	84.6% (22)	
**Smoke-free home**			
Not smoke-free home	22.1% (15)	77.9% (53)	1
Smoke-free home	27.3% (88)	72.7% (234)	0.93 (0.81,1.07)
Missing	13.8% (4)	86.2% (25)	
**SOCIODEMOGRAPHIC**			
**Current Situation Most of the Time (Study/Work/Family)**			
At School/Uni/TAFE	26.6% (38)	73.4% (105)	1
Working (not studying)	25.9% (22)	74.1% (63)	1.02 (0.86,1.21)
Parent/carer of child	25.0% (19)	75.0% (57)	1.06 (0.86,1.29)
Missing	24.4% (28)	75.6% (87)	
**Currently employed**			

Not currently employed	22.4% (66)	77.6% (229)	1
Currently employed	33.0% (37)	67.0% (75)	0.86 (0.75,0.99)*
Missing	33.3% (4)	66.7% (8)	
**Income in the past 2 weeks (AUD$) [currently working]**			
Less than $600	31.8% (14)	68.2% (30)	1
$600+	39.2% (20)	60.8% (31)	0.71 (0.52,0.97)*
Missing	17.7% (3)	82.4% (14)	
**Financial stress - any event in past 12 months**			
Ever	26.8% (77)	73.2% (210)	1
Never	26.9% (25)	73.1% (68)	0.99 (0.86,1.14)
Missing	12.8% (5)	87.2% (34)	
**Number of house moves in previous 5yrs (0-1/2-3/4+)**			
4 or more times	23.7% (18)	76.3% (58)	1
2-3 times	30.4% (51)	69.6% (117)	0.91 (0.78,1.06)
Have not moved / only once	21.2% (24)	78.8% (89)	1.04 (0.88,1.21)
Missing	22.6% (14)	77.4% (48)	
**Money to spend on self each week**			
$60 or less	26.4% (29)	73.6% (81)	1
More than $60	21.0% (22)	79.0% (83)	1.07 (0.91,1.24)
Missing	27.5% (56)	72.5% (148)	
**School attendance**			
Don't go everyday	23.8% (5)	76.2% (16)	1
Everyday	16.9% (12)	83.1% (59)	1.09 (0.83,1.42)
Missing	27.5% (90)	72.5% (237)	
**Encouragement from parents/carers/family to attend school**			
None / A little	33.3% (4)	66.7% (8)	1
Some / A lot	26.5% (30)	73.5% (83)	1.09 (0.72,1.64)
Missing	24.8% (73)	75.2% (221)	
**Expectations in 5 Years: Working full-time**			
No	19.6% (42)	80.4% (172)	1
Yes	31.7% (65)	68.3% (140)	0.85 (0.76,0.95)*
Missing	0% (0)	0% (0)	
**Amount of activities available in Community**			
None / A Little	28.3% (39)	71.7% (99)	1
Some / A Lot	26.2% (56)	71.8% (158)	1.03 (0.90,1.18)

Missing	17.9% (12)	82.1% (55)

*p<0.05 ^PR adjusted for site and age, except Age Group which is adjusted for site only.

Supplementary Table 2: Relationship between physical, mental health and cultural factors and never vaping

	Ever Used E-cigarettes % (n)	Never Used E-cigarettes % (n)	Never Used E-cigarettes PR Adj^ (95%CI)
**PHYSICAL HEALTH**			
**Smoked cigarettes**			
Ever	44.5% (94)	55.5% (117)	1
Never	5.4% (11)	94.6% (191)	1.78 (1.56,2.04)*
Missing	33.3% (2)	66.7% (4)	
**Had a full serve of alcohol**			
Ever	36.1% (84)	63.9% (149)	1
Never	32.5% (13)	67.5% (27)	1.06 (0.84,1.35)
Missing	6.9% (10)	93.2% (136)	
**Used cannabis**			
Ever	51.6% (81)	48.4% (76)	1
Never	8.3% (21)	91.7% (231)	1.89 (1.60,2.24)*
Missing	50.0% (5)	50.0% (5)	
**Relationship status**			
In a relationship	24.5% (37)	75.5% (114)	1
Not in a relationship	26.8% (61)	73.2% (167)	0.97 (0.86,1.10)
Missing	22.5% (9)	77.5% (31)	
**Sexually Active**			
Ever	36.7% (69)	63.3% (119)	1
Never	12.1% (13)	87.9% (94)	1.40 (1.23,1.60)*
Missing	20.2% (25)	79.8% (99)	
**Self-rated health status**			
Poor to Fair	34.7% (33)	65.3% (62)	1
Good to Excellent	23.8% (72)	76.2% (230)	1.15 (0.97,1.35)
Missing	9.1% (2)	90.9% (20)	
**Days physically active per week**			
0-4 days	26.5% (71)	73.5% (197)	1
5-7 days	24.1% (14)	75.9% (44)	1.03 (0.87,1.22)
Missing	23.7% (22)	76.3% (71)	
**Screen time (hrs/day) M-F (PR continuous measure)**			

Increasing time 1hr/day (0-5+) (Mean (SD))	3.6 (1.6)	3.1 (1.6)	0.96 (0.93,>1.00)
**Screen time (hrs/day) weekend (PR continuous measure)**			
Increasing time 1hr/day (0-5+) (Mean (SD))	3.5 (1.6)	3.2 (1.7)	0.97 (0.94,1.01)
**MENTAL HEALTH**			
**K5 Total Distress Score**			
High or Very High Distress (12-25)	32.2% (56)	67.8% (118)	1
Low or Moderate Distress (5-11)	21.0% (47)	79.0% (177)	1.15 (1.01,1.30)*
Missing	19.0% (4)	81.0% (17)	
**Depression diagnosis**			
Ever	36.8% (32)	63.2% (55)	1
Never	22.0% (69)	78.0% (244)	1.21 (1.01,1.46)*
Missing	31.6% (6)	68.4% (13)	
**Anxiety diagnosis**			
Ever	40.4% (38)	59.6% (56)	1
Never	20.7% (64)	79.3% (245)	1.31 (1.08,1.57)*
Missing	31.2% (5)	68.8% (11)	
**Youth Resilience Score (CYRM-12)**			
Increasing 1 unit score (0-24) (Mean (SD))	18.4 (4.7)	18.5 (4.8)	<1.00 (0.98,1.02)
Low Resilience (0-19)	25.9% (21)	74.1% (60)	1
High Resilience (20-24)	23.5% (19)	76.5% (62)	1.03 (0.87,1.23)
Missing	26.1% (67)	73.9% (190)	
**CULTURAL FACTORS**			
**Importance of Aboriginal culture**			
Not very/somewhat important	34.6% (9)	65.4% (17)	1
Important/very important	25.0% (96)	75.0% (288)	1.14 (0.85,1.53)
Missing	22.2% (2)	77.8% (7)	
**Cultural Identity:** "My connection to the land of my ancestors helps me know who I am"			
Strongly disagree-neither agree/disagree	30.3% (10)	69.7% (23)	1
At least agree/strongly agree	25.1% (95)	74.9% (283)	1.05 (0.83,1.33)
Missing	25.0% (2)	75.0% (6)	

**Culture gives confidence:** "The more I learn about my culture, the more confident I feel about my life"			
Strongly disagree-neither agree/disagree	34.0% (16)	66.0% (31)	1
At least agree/strongly agree	24.5% (89)	75.5% (275)	1.14 (0.91,1.42)
Missing	25.0% (2)	75.0% (6)	
**Speak an Indigenous language, Aboriginal English or Pidgin**			
No	25.5% (66)	74.5% (193)	1
Yes	24.3% (36)	75.7% (112)	0.99 (0.88,1.12)
Missing	41.7% (5)	58.3% (7)	
**Language is important**			
Strongly disagree-neither agree/disagree	26.7% (23)	73.3% (63)	1
At least agree/strongly agree	25.1% (80)	74.9% (239)	1.01 (0.88,1.17)
Missing	28.6% (4)	71.4% (10)	

*p<0.05 ^PR adjusted for site and age

Supplementary Table 3: Relationship between systems of exclusion and never vaping

	Ever Used E-cigarettes % (n)	Never Used E-cigarettes % (n)	Never Used E-cigarettes PR Adj^ (95%CI)
**Bullied ever**			
Yes	32.1% (43)	67.9% (91)	1
No/Never	23.0% (55)	77.0% (184)	1.12 (0.98, 1.29)
Missing	19.6% (9)	80.4% (37)	
**RACISM - OWN EXPERIENCES**			
**Racism - called insulting names**			
Yes	28.7% (49)	71.3% (122)	1
No	23.1% (48)	76.9% (160)	1.07 (0.94,1.21)
Missing	25.0% (10)	75.0% (30)	
**Racism - left out of activities**			
Yes	27.1% (26)	72.9% (70)	1
No	25.3% (71)	74.7% (210)	1.01 (0.87,1.16)
Missing	23.8% (10)	76.2% (32)	
**Racism - physical abuse**			
Yes	32.7% (17)	67.3% (35)	1
No	24.5% (80)	75.5% (247)	1.11 (0.90,1.36)
Missing	25.0% (10)	75.0% (30)	
**Racism - people think didn't speak English well**			
Yes	22.9% (11)	77.1% (37)	1
No	25.9% (85)	74.1% (243)	0.97 (0.82,1.15)
Missing	25.6% (11)	74.4% (32)	
**Racism - people suspicious of you**			
Yes	32.4% (46)	67.6% (96)	1
No	21.3% (50)	78.7% (185)	1.16 (1.01,1.33)*
Missing	26.2% (11)	73.8% (31)	
**Racism - poor service at a restaurant/food outlet**			
Yes	28.2% (29)	71.8% (74)	1
No	24.6% (67)	75.4% (205)	1.05 (0.91,1.20)
Missing	25.0% (11)	75.0% (33)	
**Racism - treated badly in a shop**			
Yes	31.6% (50)	68.4% (108)	1

No	21.1% (47)	78.9% (176)	1.16 (1.02,1.32)*
Missing	26.3% (10)	73.7% (28)	
**Racism - put in a lower ability class or group**			
Yes	30.9% (21)	69.1% (47)	1
No	26.3% (62)	73.7% (174)	1.07 (0.89,1.29)
Missing	20.9% (24)	79.1% (91)	
**Racism - disciplined unfairly or given school detention**			
Yes	31.0% (26)	69.0% (58)	1
No	25.8% (56)	74.2% (161)	1.08 (0.92,1.28)
Missing	21.2% (25)	78.8% (93)	
**Racism - given a lower grade or mark than you deserved**			
Yes	30.8% (16)	69.2% (36)	1
No	26.8% (67)	73.2% (183)	1.06 (0.87,1.29)
Missing	20.5% (24)	79.5% (93)	
**Racism - hassled by the police**			
Yes	40.8% (49)	59.2% (71)	1
No	18.6% (48)	81.4% (210)	1.38 (1.17,1.62)*
Missing	24.4% (10)	75.6% (31)	
**RACISM - ANY (of all questions)**			
Experienced at least 1 event	30.2% (78)	69.8% (180)	1
Not experienced any (or did not answer)	15.0% (19)	85.0% (108)	1.21 (1.08,1.36)*
Missing	29.4% (10)	70.6% (24)	
**RACISM - VICARIOUS RACISM EXPERIENCES**			
**Vicarious Racism - ever seen someone treated badly**			
Ever	27.6% (85)	72.4% (223)	1
Never	20.0% (15)	80.0% (60)	1.12 (0.98,1.28)
Missing	19.4% (7)	80.6% (29)	
**Vicarious Racism - ever seen someone left out**			
Ever	27.3% (80)	72.7% (213)	1
Never	21.0% (17)	79.0% (64)	1.10 (0.96,1.26)
Missing	22.2% (10)	77.8% (35)	
**Vicarious Racism - ever seen someone called names**			
Ever	27.5% (83)	72.5% (219)	1
Never	20.5% (16)	79.5% (62)	1.10 (0.96,1.26)

Missing	20.5% (8)	79.5% (31)	
**Vicarious Racism - ever seen someone physically abused**			
Ever	26.0% (67)	74.0% (191)	1
Never	26.8% (33)	73.2% (90)	<1.00 (0.87,1.13)
Missing	18.4% (7)	81.6% (31)	
**Vicarious Racism - negative media portrayal**			
Ever	29.0% (81)	71.0% (198)	1
Never	13.0% (10)	87.0% (67)	1.24 (1.10,1.39)*
Missing	25.4% (16)	74.6% (47)	
**VICARIOUS RACISM - ANY**			
Ever experienced any vicarious racism	27.1% (90)	72.9% (242)	1
Never	17.9% (10)	82.1% (46)	1.14 (0.99,1.31)
Missing	22.6% (7)	77.4% (24)	
**JUSTICE SYSTEM INTERACTIONS**			
**Police - ever questioned/given warning**			
Yes	36.0% (54)	64.0% (96)	1
No	18.7% (40)	81.3% (174)	1.26 (1.10,1.44)*
Missing	23.6% (13)	76.4% (42)	
**Police - ever harassed (physical/verbal abuse)**			
Yes	44.4% (8)	55.6% (10)	1
No	12.7% (7)	87.3% (48)	1.53 (1.02,2.29)*
Missing	26.6% (92)	73.4% (254)	
**Police - ever charged**			
Yes	34.2% (25)	65.8% (48)	1
No	25.3% (74)	74.7% (219)	1.13 (0.94,1.36)
Missing	15.1% (8)	84.9% (45)	
**Police - ever sent to jail by a judge/magistrate**			
Yes	36.0% (9)	64.0% (16)	1
No	26.4% (90)	73.6% (251)	1.15 (0.85,1.54)
Missing	15.1% (8)	84.9% (45)	
**Police - have friends who have been to jail**			
Yes	32.9% (47)	67.1% (96)	1
No	21.5% (46)	78.5% (168)	1.16 (1.02,1.33)*
Missing	22.6% (14)	77.4% (48)	

**Ever had interactions with the justice system (combined)**			
Ever	34.7% (68)	65.3% (128)	1
Never	17.5% (31)	82.5% (146)	1.25 (1.11,1.41)*
Missing	17.4% (8)	82.6% (38)	

*p<0.05 ^PR adjusted for site and age
