# Supplementary material for: Protective Factors Against e‐Cigarette Use Among First Nations People Aged 16–24 in the Next Generation Youth Wellbeing Study
Source: Health Promot J Austr. 2025 Feb 6;36(2):e951. doi: 10.1002/hpja.951 (PMC11926581; doi:10.1002/hpja.951)
Supplement: Supplementary file 2 — Table S1.Relationship between demographic/sociodemographic factors and never vaping. Table S2. Relationship between physical, mental health and cultural factors and never vaping. Table S3. Relationship between systems of exclusion and never vaping. [file HPJA-36-0-s002.docx]

**Supplementary Table 1: Relationship between demographic/sociodemographic factors and never vaping**

|  | **Ever Used**  **E-cigarettes**  **% (n)** | **Never Used**  **E-cigarettes**  **% (n)** | **Never Used**  **E-cigarettes**  **PR Adj^ (95%CI)** |
| --- | --- | --- | --- |
| **DEMOGRAPHIC** | | | |
|  |  |  |  |
| **Site** | | | |
|  |  |  |  |
| New South Wales | 28.9% (41) | 71.1% (101) | 1 |
| Central Australia | 9.5% (4) | 90.5% (38) | 1.28 (1.11,1.47)* |
| Western Australia | 26.4% (62) | 73.6% (173) | 1.03 (0.91,1.18) |
| Missing | 0% (0) | 0% (0) |  |
|  |  |  |  |
| **Sex** | | | |
| Female | 24.8% (66) | 75.2% (200) | 1 |
| Male | 27.6% (40) | 72.4% (105) | 0.95 (0.84,1.07) |
| Missing | 12.5% (1) | 87.5% (7) |  |
|  |  |  |  |
| **Age group** | | | |
| 16-17 | 23.0% (34) | 77.0% (114) | 1 |
| 18-24 | 26.9% (73) | 73.1% (198) | 0.94 (0.84,1.05) |
| Missing | 0% | 0% |  |
|  |  |  |  |
| **Friends who smoke** | | | |
| Have smoking friends | 31.8% (98) | 68.2% (210) | 1 |
| Non-smoking friends | 5.9% (5) | 94.1% (80) | 1.38 (1.26,1.51)* |
| Missing | 15.4% (4) | 84.6% (22) |  |
|  |  |  |  |
| **Smoke-free home** | | | |
| Not smoke-free home | 22.1% (15) | 77.9% (53) | 1 |
| Smoke-free home | 27.3% (88) | 72.7% (234) | 0.93 (0.81,1.07) |
| Missing | 13.8% (4) | 86.2% (25) |  |
|  |  |  |  |
| **SOCIODEMOGRAPHIC** | | | |
|  | | | |
| **Current Situation Most of the Time (Study/Work/Family)** | | | |
| At School/Uni/TAFE | 26.6% (38) | 73.4% (105) | 1 |
| Working (not studying) | 25.9% (22) | 74.1% (63) | 1.02 (0.86,1.21) |
| Parent/carer of child | 25.0% (19) | 75.0% (57) | 1.06 (0.86,1.29) |
| Missing | 24.4% (28) | 75.6% (87) |  |
|  | | | |
| **Currently employed** | | | |
| Not currently employed | 22.4% (66) | 77.6% (229) | 1 |
| Currently employed | 33.0% (37) | 67.0% (75) | 0.86 (0.75,0.99)* |
| Missing | 33.3% (4) | 66.7% (8) |  |
|  | | | |
| **Income in the past 2 weeks (AUD$) [currently working]** | | | |
| Less than $600 | 31.8% (14) | 68.2% (30) | 1 |
| $600+ | 39.2% (20) | 60.8% (31) | 0.71 (0.52,0.97)* |
| Missing | 17.7% (3) | 82.4% (14) |  |
|  | | | |
| **Financial stress - any event in past 12 months** | | | |
| Ever | 26.8% (77) | 73.2% (210) | 1 |
| Never | 26.9% (25) | 73.1% (68) | 0.99 (0.86,1.14) |
| Missing | 12.8% (5) | 87.2% (34) |  |
|  | | | |
| **Number of house moves in previous 5yrs (0-1/2-3/4+)** | | | |
| 4 or more times | 23.7% (18) | 76.3% (58) | 1 |
| 2-3 times | 30.4% (51) | 69.6% (117) | 0.91 (0.78,1.06) |
| Have not moved / only once | 21.2% (24) | 78.8% (89) | 1.04 (0.88,1.21) |
| Missing | 22.6% (14) | 77.4% (48) |  |
|  | | | |
| **Money to spend on self each week** | | | |
| $60 or less | 26.4% (29) | 73.6% (81) | 1 |
| More than $60 | 21.0% (22) | 79.0% (83) | 1.07 (0.91,1.24) |
| Missing | 27.5% (56) | 72.5% (148) |  |
|  | | | |
| **School attendance** | | | |
| Don't go everyday | 23.8% (5) | 76.2% (16) | 1 |
| Everyday | 16.9% (12) | 83.1% (59) | 1.09 (0.83,1.42) |
| Missing | 27.5% (90) | 72.5% (237) |  |
|  | | | |
| **Encouragement from parents/carers/family to attend school** | | | |
| None / A little | 33.3% (4) | 66.7% (8) | 1 |
| Some / A lot | 26.5% (30) | 73.5% (83) | 1.09 (0.72,1.64) |
| Missing | 24.8% (73) | 75.2% (221) |  |
|  | | | |
| **Expectations in 5 Years: Working full-time** | | | |
| No | 19.6% (42) | 80.4% (172) | 1 |
| Yes | 31.7% (65) | 68.3% (140) | 0.85 (0.76,0.95)* |
| Missing | 0% (0) | 0% (0) |  |
|  | | | |
| **Amount of activities available in Community** | | | |
| None / A Little | 28.3% (39) | 71.7% (99) | 1 |
| Some / A Lot | 26.2% (56) | 71.8% (158) | 1.03 (0.90,1.18) |
| Missing | 17.9% (12) | 82.1% (55) |  |

*p<0.05 ^PR adjusted for site and age, except Age Group which is adjusted for site only.

**Supplementary Table 2: Relationship between physical, mental health and cultural factors and never vaping**

|  | **Ever Used**  **E-cigarettes**  **% (n)** | **Never Used**  **E-cigarettes**  **% (n)** | **Never Used**  **E-cigarettes**  **PR Adj^ (95%CI)** |
| --- | --- | --- | --- |
| **PHYSICAL HEALTH** | | | |
|  | | | |
| **Smoked cigarettes** | | | |
| Ever | 44.5% (94) | 55.5% (117) | 1 |
| Never | 5.4% (11) | 94.6% (191) | 1.78 (1.56,2.04)* |
| Missing | 33.3% (2) | 66.7% (4) |  |
|  | | | |
| **Had a full serve of alcohol** | | | |
| Ever | 36.1% (84) | 63.9% (149) | 1 |
| Never | 32.5% (13) | 67.5% (27) | 1.06 (0.84,1.35) |
| Missing | 6.9% (10) | 93.2% (136) |  |
|  | | | |
| **Used cannabis** | | | |
| Ever | 51.6% (81) | 48.4% (76) | 1 |
| Never | 8.3% (21) | 91.7% (231) | 1.89 (1.60,2.24)* |
| Missing | 50.0% (5) | 50.0% (5) |  |
|  | | | |
| **Relationship status** | | | |
| In a relationship | 24.5% (37) | 75.5% (114) | 1 |
| Not in a relationship | 26.8% (61) | 73.2% (167) | 0.97 (0.86,1.10) |
| Missing | 22.5% (9) | 77.5% (31) |  |
|  | | | |
| **Sexually Active** | | | |
| Ever | 36.7% (69) | 63.3% (119) | 1 |
| Never | 12.1% (13) | 87.9% (94) | 1.40 (1.23,1.60)* |
| Missing | 20.2% (25) | 79.8% (99) |  |
|  | | | |
| **Self-rated health status** | | | |
| Poor to Fair | 34.7% (33) | 65.3% (62) | 1 |
| Good to Excellent | 23.8% (72) | 76.2% (230) | 1.15 (0.97,1.35) |
| Missing | 9.1% (2) | 90.9% (20) |  |
|  | | | |
| **Days physically active per week** | | | |
| 0-4 days | 26.5% (71) | 73.5% (197) | 1 |
| 5-7 days | 24.1% (14) | 75.9% (44) | 1.03 (0.87,1.22) |
| Missing | 23.7% (22) | 76.3% (71) |  |
|  | | | |
| **Screen time (hrs/day) M-F (PR continuous measure)** | | | |
| Increasing time 1hr/day (0-5+) (Mean (SD)) | 3.6 (1.6) | 3.1 (1.6) | 0.96 (0.93,>1.00) |
|  | | | |
| **Screen time (hrs/day) weekend (PR continuous measure)** | | | |
| Increasing time 1hr/day (0-5+) (Mean (SD)) | 3.5 (1.6) | 3.2 (1.7) | 0.97 (0.94,1.01) |
|  |  |  |  |
| **MENTAL HEALTH** | | | |
|  | | | |
| **K5 Total Distress Score** | | | |
| High or Very High Distress (12-25) | 32.2% (56) | 67.8% (118) | 1 |
| Low or Moderate Distress (5-11) | 21.0% (47) | 79.0% (177) | 1.15 (1.01,1.30)* |
| Missing | 19.0% (4) | 81.0% (17) |  |
|  | | | |
| **Depression diagnosis** | | | |
| Ever | 36.8% (32) | 63.2% (55) | 1 |
| Never | 22.0% (69) | 78.0% (244) | 1.21 (1.01,1.46)* |
| Missing | 31.6% (6) | 68.4% (13) |  |
|  | | | |
| **Anxiety diagnosis** | | | |
| Ever | 40.4% (38) | 59.6% (56) | 1 |
| Never | 20.7% (64) | 79.3% (245) | 1.31 (1.08,1.57)* |
| Missing | 31.2% (5) | 68.8% (11) |  |
|  | | | |
| **Youth Resilience Score (CYRM-12)** | | | |
| Increasing 1 unit score (0-24) (Mean (SD)) | 18.4 (4.7) | 18.5 (4.8) | <1.00 (0.98,1.02) |
|  |  |  |  |
| Low Resilience (0-19) | 25.9% (21) | 74.1% (60) | 1 |
| High Resilience (20-24) | 23.5% (19) | 76.5% (62) | 1.03 (0.87,1.23) |
| Missing | 26.1% (67) | 73.9% (190) |  |
|  | | | |
| **CULTURAL FACTORS** | | | |
|  | | | |
| **Importance of Aboriginal culture** | | | |
| Not very/somewhat important | 34.6% (9) | 65.4% (17) | 1 |
| Important/very important | 25.0% (96) | 75.0% (288) | 1.14 (0.85,1.53) |
| Missing | 22.2% (2) | 77.8% (7) |  |
|  | | | |
| **Cultural Identity:** “My connection to the land of my ancestors helps me know who I am” | | | |
| Strongly disagree-neither agree/disagree | 30.3% (10) | 69.7% (23) | 1 |
| At least agree/strongly agree | 25.1% (95) | 74.9% (283) | 1.05 (0.83,1.33) |
| Missing | 25.0% (2) | 75.0% (6) |  |
|  | | | |
| **Culture gives confidence:** “The more I learn about my culture, the more confident I feel about my life” | | | |
| Strongly disagree-neither agree/disagree | 34.0% (16) | 66.0% (31) | 1 |
| At least agree/strongly agree | 24.5% (89) | 75.5% (275) | 1.14 (0.91,1.42) |
| Missing | 25.0% (2) | 75.0% (6) |  |
|  | | | |
| **Speak an Indigenous language, Aboriginal English or Pidgin** | | | |
| No | 25.5% (66) | 74.5% (193) | 1 |
| Yes | 24.3% (36) | 75.7% (112) | 0.99 (0.88,1.12) |
| Missing | 41.7% (5) | 58.3% (7) |  |
|  | | | |
| **Language is important** | | | |
| Strongly disagree-neither agree/disagree | 26.7% (23) | 73.3% (63) | 1 |
| At least agree/strongly agree | 25.1% (80) | 74.9% (239) | 1.01 (0.88,1.17) |
| Missing | 28.6% (4) | 71.4% (10) |  |

*p<0.05 ^PR adjusted for site and age

**Supplementary Table 3: Relationship between systems of exclusion and never vaping**

|  | **Ever Used**  **E-cigarettes**  **% (n)** | **Never Used**  **E-cigarettes**  **% (n)** | **Never Used**  **E-cigarettes**  **PR Adj^ (95%CI)** |
| --- | --- | --- | --- |
|  | | | |
| **Bullied ever** | | | |
| Yes | 32.1% (43) | 67.9% (91) | 1 |
| No/Never | 23.0% (55) | 77.0% (184) | 1.12 (0.98, 1.29) |
| Missing | 19.6% (9) | 80.4% (37) |  |
|  |  |  |  |
| **RACISM - OWN EXPERIENCES** | | | |
|  | | | |
| **Racism - called insulting names** | | | |
| Yes | 28.7% (49) | 71.3% (122) | 1 |
| No | 23.1% (48) | 76.9% (160) | 1.07 (0.94,1.21) |
| Missing | 25.0% (10) | 75.0% (30) |  |
|  |  |  |  |
| **Racism - left out of activities** | | | |
| Yes | 27.1% (26) | 72.9% (70) | 1 |
| No | 25.3% (71) | 74.7% (210) | 1.01 (0.87,1.16) |
| Missing | 23.8% (10) | 76.2% (32) |  |
|  | | | |
| **Racism - physical abuse** | | | |
| Yes | 32.7% (17) | 67.3% (35) | 1 |
| No | 24.5% (80) | 75.5% (247) | 1.11 (0.90,1.36) |
| Missing | 25.0% (10) | 75.0% (30) |  |
|  | | | |
| **Racism - people think didn't speak English well** | | | |
| Yes | 22.9% (11) | 77.1% (37) | 1 |
| No | 25.9% (85) | 74.1% (243) | 0.97 (0.82,1.15) |
| Missing | 25.6% (11) | 74.4% (32) |  |
|  | | | |
| **Racism - people suspicious of you** | | | |
| Yes | 32.4% (46) | 67.6% (96) | 1 |
| No | 21.3% (50) | 78.7% (185) | 1.16 (1.01,1.33)* |
| Missing | 26.2% (11) | 73.8% (31) |  |
|  | | | |
| **Racism - poor service at a restaurant/food outlet** | | | |
| Yes | 28.2% (29) | 71.8% (74) | 1 |
| No | 24.6% (67) | 75.4% (205) | 1.05 (0.91,1.20) |
| Missing | 25.0% (11) | 75.0% (33) |  |
|  | | | |
| **Racism - treated badly in a shop** | | | |
| Yes | 31.6% (50) | 68.4% (108) | 1 |
| No | 21.1% (47) | 78.9% (176) | 1.16 (1.02,1.32)* |
| Missing | 26.3% (10) | 73.7% (28) |  |
|  | | | |
| **Racism - put in a lower ability class or group** | | | |
| Yes | 30.9% (21) | 69.1% (47) | 1 |
| No | 26.3% (62) | 73.7% (174) | 1.07 (0.89,1.29) |
| Missing | 20.9% (24) | 79.1% (91) |  |
|  | | | |
| **Racism - disciplined unfairly or given school detention** | | | |
| Yes | 31.0% (26) | 69.0% (58) | 1 |
| No | 25.8% (56) | 74.2% (161) | 1.08 (0.92,1.28) |
| Missing | 21.2% (25) | 78.8% (93) |  |
|  | | | |
| **Racism - given a lower grade or mark than you deserved** | | | |
| Yes | 30.8% (16) | 69.2% (36) | 1 |
| No | 26.8% (67) | 73.2% (183) | 1.06 (0.87,1.29) |
| Missing | 20.5% (24) | 79.5% (93) |  |
|  | | | |
| **Racism - hassled by the police** | | | |
| Yes | 40.8% (49) | 59.2% (71) | 1 |
| No | 18.6% (48) | 81.4% (210) | 1.38 (1.17,1.62)* |
| Missing | 24.4% (10) | 75.6% (31) |  |
|  | | | |
| **RACISM - ANY (of all questions)** | | | |
| Experienced at least 1 event | 30.2% (78) | 69.8% (180) | 1 |
| Not experienced any (or did not answer) | 15.0% (19) | 85.0% (108) | 1.21 (1.08,1.36)* |
| Missing | 29.4% (10) | 70.6% (24) |  |
|  |  |  |  |
| **RACISM - VICARIOUS RACISM EXPERIENCES** | | | |
|  | | | |
| **Vicarious Racism - ever seen someone treated badly** | | | |
| Ever | 27.6% (85) | 72.4% (223) | 1 |
| Never | 20.0% (15) | 80.0% (60) | 1.12 (0.98,1.28) |
| Missing | 19.4% (7) | 80.6% (29) |  |
|  | | | |
| **Vicarious Racism - ever seen someone left out** | | | |
| Ever | 27.3% (80) | 72.7% (213) | 1 |
| Never | 21.0% (17) | 79.0% (64) | 1.10 (0.96,1.26) |
| Missing | 22.2% (10) | 77.8% (35) |  |
|  |  |  |  |
| **Vicarious Racism - ever seen someone called names** | | | |
| Ever | 27.5% (83) | 72.5% (219) | 1 |
| Never | 20.5% (16) | 79.5% (62) | 1.10 (0.96,1.26) |
| Missing | 20.5% (8) | 79.5% (31) |  |
|  | | | |
| **Vicarious Racism - ever seen someone physically abused** | | | |
| Ever | 26.0% (67) | 74.0% (191) | 1 |
| Never | 26.8% (33) | 73.2% (90) | <1.00 (0.87,1.13) |
| Missing | 18.4% (7) | 81.6% (31) |  |
|  | | | |
| **Vicarious Racism - negative media portrayal** | | | |
| Ever | 29.0% (81) | 71.0% (198) | 1 |
| Never | 13.0% (10) | 87.0% (67) | 1.24 (1.10,1.39)* |
| Missing | 25.4% (16) | 74.6% (47) |  |
|  | | | |
| **VICARIOUS RACISM - ANY** | | | |
| Ever experienced any vicarious racism | 27.1% (90) | 72.9% (242) | 1 |
| Never | 17.9% (10) | 82.1% (46) | 1.14 (0.99,1.31) |
| Missing | 22.6% (7) | 77.4% (24) |  |
|  |  |  |  |
| **JUSTICE SYSTEM INTERACTIONS** | | | |
|  | | | |
| **Police - ever questioned/given warning** | | | |
| Yes | 36.0% (54) | 64.0% (96) | 1 |
| No | 18.7% (40) | 81.3% (174) | 1.26 (1.10,1.44)* |
| Missing | 23.6% (13) | 76.4% (42) |  |
|  | | | |
| **Police - ever harassed (physical/verbal abuse)** | | | |
| Yes | 44.4% (8) | 55.6% (10) | 1 |
| No | 12.7% (7) | 87.3% (48) | 1.53 (1.02,2.29)* |
| Missing | 26.6% (92) | 73.4% (254) |  |
|  | | | |
| **Police - ever charged** | | | |
| Yes | 34.2% (25) | 65.8% (48) | 1 |
| No | 25.3% (74) | 74.7% (219) | 1.13 (0.94,1.36) |
| Missing | 15.1% (8) | 84.9% (45) |  |
|  | | | |
| **Police - ever sent to jail by a judge/magistrate** | | | |
| Yes | 36.0% (9) | 64.0% (16) | 1 |
| No | 26.4% (90) | 73.6% (251) | 1.15 (0.85,1.54) |
| Missing | 15.1% (8) | 84.9% (45) |  |
|  | | | |
| **Police - have friends who have been to jail** | | | |
| Yes | 32.9% (47) | 67.1% (96) | 1 |
| No | 21.5% (46) | 78.5% (168) | 1.16 (1.02,1.33)* |
| Missing | 22.6% (14) | 77.4% (48) |  |
|  | | | |
| **Ever had interactions with the justice system (combined)** | | | |
| Ever | 34.7% (68) | 65.3% (128) | 1 |
| Never | 17.5% (31) | 82.5% (146) | 1.25 (1.11,1.41)* |
| Missing | 17.4% (8) | 82.6% (38) |  |

*p<0.05 ^PR adjusted for site and age
